# A VALUES-DRIVEN APPROACH TO BRIDGING THE DIGITAL DIVIDE: A PILOT INTERVENTION FOR OLDER ADULTS

**DOI:** 10.1093/geroni/igae098.2116

**Published:** 2024-12-31

**Authors:** Melissa Hladek, Olivia Rubio, Kennedy McDaniel, Allyson Evelyn-Gustave, Deidra Crews, Mara McAdams DeMarco, Daniel Brennan, Sarah Szanton

**Affiliations:** Johns Hopkins University, Johns Hopkins University, Maryland, United States; The Pennsylvania State University, University Park, Pennsylvania, United States; Johns Hopkins University, Baltimore, Maryland, United States; Johns Hopkins University, Baltimore, Maryland, United States; Johns Hopkins University, Baltimore, Maryland, United States; New York University, New York, New York, United States; Johns Hopkins University School of Medicine, Baltimore, Maryland, United States; Johns Hopkins University, Baltimore, Maryland, United States

## Abstract

Digital literacy, the ability to effectively use technology for information retrieval, evaluation, and communication, is critical in today’s world. Despite advancements, older adults lag behind younger generations with approximately one in four adults over 65 not using the internet and over 35% lacking broadband connectivity. This gap is exacerbated among older adults of color due to disparities in access, connectivity, affordability, and cultural factors. Recognizing older adults’ diverse values and learning styles, we propose a novel intervention tailored to individual values and goals. This one-arm feasibility and acceptability pilot trial enrolls 20 multi-racial older adults with varying levels of digital literacy, delivering a person-directed, values-based intervention in their homes over 4-6 visits. Our intervention targets community-identified barriers such as health literacy, health communication, and social support, aiming to enhance digital literacy with additional outcomes including quality of life and frailty. We view internet access and training not only as means to improve digital literacy but also as vital tools in addressing health disparities. By leveraging individual values and theoretically driven strategies, this intervention tests a potential avenue to narrow the digital divide among older adults, fostering inclusion, empowerment, and improved health outcomes.

